# Mississippi Valley Association of Dental Surgeons

**Published:** 1887-04

**Authors:** 


### Mississippi Valley Association of Dental Surgeons.

(Reported for the Register.)

### HISTORY OF THE ASSOCIATION.

(See Paper by Dr. Betty, published in March Register, page 131.)

### DISCUSSION.

Cincinnati, March. 2d, 3d, and 4th, 1887.

*Dr. J. Taft.*—The prevailing spirit then, more so than now, was against the patenting of any appliance or article used by the profession. There was a stronger feeling against anything that seemed not to be correct—especially so in regard to ethics and the amalgam question. It was, perhaps, first in this body that sentiments strongly antagonistic to the use of amalgam were expressed. Phases of society work, having their origin here, have left their mark on the more than one hundred societies now existing—have permeated and been a stimulus to the profession throughout the country.

*Dr. Geo. W. Keely.*—An incident happened in this room that made a great impression on me. It was away back in the "forties" (prior to which plates were made to be held in place by means of spiral springs, and I ventured the assertion that a plate so made was never worn with comfort), when Dr. Goddard undertook to insert plates by atmospheric pressure or suction. He put them in the patient's mouth; the upper plate fell down and the lower one kicked up. Told the patient "all right," "now open your mouth," "now close your mouth," "all right." "Got a handkerchief?" "Give it to me." And he tied up the patient's jaws directing her to go home and take out the plates when eating, but to put them back immediately after eating and tie up the head as before. Coming back in a few days, as he had directed, he removed the bandage and the plates were as loose as before. With an "all right" he tied up the jaws again and sent the patient home for another few days. After a few week's persistence and some changes made in the plates, they fitted very comfortably and the patient was dismissed.

*Dr. H. A. Smith.*—Dentistry is a very exacting and confining profession and my only pleasurable recollection of it is the social intercourse at dental meetings. If I were to leave out the pleasures of my connection with dental associations, there would be very little left. I can not understand why every dentist is not a society man. They certainly owe it to themselves and their patients to advance with the profession.

*Dr. James Leslie.*—The best amalgam alloy, cohesive gold-foil, and continuous gum-work, were all first introduced in this association and I have the papers to prove it.

*Dr. C. M. Wright.*—I wish to speak of its influence on the world as a missionary society. Its members have gone to all parts of the world and three out of the four dentists who established the American Dental Society of Europe were members of this association.

*Dr. H. J. McKellops.*—In 1864 I represented this society in London, and there and in Paris first introduced the hand mallet.

(See Dr. Wright's paper on Nutrition, page 169)

DISCUSSION.

*Dr. A. O. Rawls.*—I take it that this subject involves replantation and implantation of the teeth. I do not believe the pulp to be the only source of nutrition to the teeth. There is a supply by means of anastomosis and endosmotic action that might keep the parts tolerant to the tooth; but I apprehend there is not a return of the nutrient supply after the endomosis is interrupted or broken up—if you break the continuity you more or less deprive the tooth of its proportion of the nutrient supply.

*Dr. H. A. Smith.*—Dr. Wright said when a tooth is attacked by caries it affected the entire system. If it is true that there is a reparative action set up in a carious tooth, it is so inappreciable as not to be noticed by the ordinary practitioner. I believe Dr. Wright takes the position that in the adult the quantity of lime in the teeth is no greater than in the teeth of the young. Is it true or not that the teeth in middle life are more dense, or is it only the surface that is condensed? How is it below the surface? Does caries excite nutrition and carry into the teeth the inorganic matter we call lime-salts? In later life we find an excessive deposit of salivary calculus about the teeth and that decay has ceased. May that not be the excess of lime salts not needed in the nutritive functions of the teeth? Do we find in persons of adult life less dental caries than in the young?

*Dr. F. W. Sage.*—Is not the presence or absence of tartar and hardening of the teeth a mere coincidence? Is it not to be accounted for by the individual habits of those who do or do not live sedentary lives? Many pulpless teeth, without antagonists, after being treated and the canals filled to restore them to use fulness are total failures, and should have been extracted in the first place. I think many operations in crown and bridge work come under this head.

*Dr. J. Taft.*---There need not be any doubt about the corresponding conditions of nutrition in other parts of the body. If the dentist is to have the care of the child's teeth, it is to his interest as well as that of the child and its parents that the body be well nourished. It is an important and a practical point. He should pursue such a course with them as will secure a healthy condition of the whole body, and in doing so, is he not rendering just as good service as if performing operations on the teeth? Shall he accept this as only an incident and allow it to continue, or shall he try to correct it?

Professor Smith has asked the question, when a tooth is once made up, is it made up once for all without a change? There cannot be any doubt on that question, for the teeth of children are composed of a greater amount of organic material. Examined under the microscope, they show the lack of calcification, and chemical analysis proves that the teeth of old people are more dense. The enamel is dependent entirely on the internal supply of nutrition, and is devitalized when the pulp is destroyed. It is different with the cementum, which receives its nutrition from the periosteum. There is often a change in the contents of the tubuli that prevents, forever, the restoration of the periosteal tissue to a normal condition. Once this happens, I do not believe it can ever be restored.

*Dr. M. H. Fletcher* presented specimens of elephant ivory, which, he said, we know is a tooth of permanent formation. Bullets had been shot into them, and became encapsuled or encysted by a new growth of ivory or dentine, caused by the irritation. We all know that amalgam and other metallic fillings often excite the deposits of secondary dentine. I have in my possession a specimen of perverted nutrition, a tooth containing a tumor (nearly as large as the tooth), composed of a shell, or deposit of dentine. The tooth was worn down to the pulp cavity.

*Dr. A. W. Harlan.*—I will begin at the end of Dr. Wright's paper, where he advocates that a dentist should be more than a tooth filler. I consider that one of the greatest needs of the profession to-day is that we should have a wider knowledge of principles, and not confine ourselves to the mechanics and be mere tooth fillers and tooth makers. With the nihilistic ideas of the paper I cannot agree ; a pulpless tooth need not of necessity be a foreign body. I cannot agree that it would be better to pluck it out than to transplant, replant, or crown. If all pulpless teeth were extracted, a good many people would be edentulous, or nearly so, for in some mouths there are six or more teeth doing satisfactory service that have not been restored entirely to health.

*Dr. A. O. Rawls.*—And never can be.

I realize that there are many roots of teeth treated and crowned that should be extracted, but it is wrong to teach that nearly all pulpless teeth should be extracted. It requires good judgment to know when to retain or extract a tooth. . . . After a tooth has been fully formed, is erupted through the gums, and takes its place in the arch, I apprehend that it increases in density very little after the twentieth year, except for mechanical or external reasons. If its tendency to decay increases it is on account of its surroundings or environments. The deposit of secondary dentine shown by Dr. Fletcher was caused by irritation, which will undoubtedly produce this condition. But take a sound tooth in good physiological condition, and does the pulp become obliterated by excess of nutrition? There are few such cases on record. Tomes, I believe, reports one case, and Dr. Judd one. I have not been able to find twenty cases recorded, and of the millions of teeth bored into or extracted why do we not find more cases of transformation of pulp tissue, if there is this excess of nutrition? It is impossible for the inner surface of a tooth to be torn down and carried away, because the pulp has no such function.

*Dr. A. O. Rawls.*—The gentleman is entirely wrong on one point. We know that when the body is young it contains more organic material. It is not necessary that the structure be torn down to reconstruct it. All canaliculi or tubuli become closed in old age—denser and harder—mummifying the soft tissues and condensing the hard. The hard matter is added to from time to time, and if we could live long enough we would not have any soft tissues in our bodies—we would mummify. It is perfectly absurd to think of a pulpless tooth ever being restored to health, for the continuity of the anastomosis is destroyed. There is a line dividing the parts, because a hard substance is in contact with a soft tissue, and it will break down; it can not incyst, like lead or iron, but becomes a foreign substance. If the cementum is once in contact with the dead dentine, then there is a departure from health, and it becomes an irritant.

*Dr. H. J. McKellops.*—Why do they remain then?

*Dr. Rawls.*—I'll tell you why—and every one ought to know enough physiology to know it—you can not do it in the mouth of a child twelve years old.

*Dr. McKellops.*—Yes, I can. Why do fractured bones reunite when fastened with ivory points?

*Dr. Rawls.*—It is only tolerance. You can't tell why they unite. The ivory is a source of irritation which may tend to increase the flow of nutrition. In answer to the question, Is nutrition given to the tooth after it has once been formed? I think it is growing all the time, and the pulp is growing less all the time, until in old age it gradually becomes solidified, especially where they live on a diet containing a great deal of lime salts. There was a case in one of the Southern States where all the soft tissue was obliterated, and the teeth had to be pinched out piece by piece until the bottom was reached—a solid mass from the crown to the apex of the root.

*Dr. M. H. Fletcher.*—It is not unusual to find in the cementum tubuli analogous to the Haversian canals, and these tubuli can be seen running into the enamel and dentine—the dentine, cementum, and enamel connected—and may supply sufficient nutrition to keep it alive for years.

*Dr. McKellops.*—Dr. Patrick has been investigating the cause of abrasion of the teeth, and finds it in an acid secretion of the mucous follicles. No man ever saw abrasion caused by the brush or other mechanical means. Some follicles give off the acid, and others do not. You will find it by turning up the lip opposite an abrasion on the faces of the teeth, drying it, and testing for the acid.

*Dr. H. A. Smith.*—The nutrition of the teeth is involved in this erosion. If the teeth undergo a change whereby nutrition is withdrawn may that not account for it? If there is a consolidation going on from youth to old age, why not an unbuilding? There are supposed to be two kinds of disintegration—abrasion and erosion. Can we distinguish between them?

*Dr. J. Taft.*—I have specimens of teeth where the crowns were broken off, in early life, at an angle with the root and reunited at the angle. If there wasn't a nutrient supply from the pulp that couldn't have happened. Dr. Rawls said that a pulpless tooth couldn't remain without being encysted or becoming an irritant. The cement is living and is in contact with the periosteum; there is no exfoliation. A step further—suppose the cement dies; there is the dead cement attached to the periosteum and retaining the teeth in position, though not as firmly as before devitalization, but I believe the cement retains its vitality in the main so long as the tooth remains in the socket without periosteal disturbance.

*Dr. W. N. Morrison.*—I have been interested in implantation, but can't say that I think the peridental membrane retains its vitality after being out of the socket for years; but a tooth that has been replanted or transplanted does regain an outward appearance of life. We can not know the results of implantation for several years.

*Dr. McKellops.*—I can testify to the implantion I have seen. A right lateral incisor for a woman thirty years of age, a tooth that had been out two years. The tooth has been in its new socket six weeks, and has been pulled at and hauled at by about forty persons. It has been examined by an eminent surgeon and oculist. The former could not tell which tooth it was until pointed out to him. When inserted it was as white as a sheet of writing paper, but after a few days regained its natural color.

I do not like to see a man criticised by those who have never seen the operation. No sane man would perform the operations cited by Dr. Rawls in his article in the *Southern Dental Journal.*

Dr. Younger is that kind of a man—he doesn't take cases that he is not satisfied he can succeed with. He selects his cases carefully. You must have a favorable case. Do you suppose you can take an old man, with a jaw like a sucking babe's, and implant a full set of teeth in it? Dr. Younger is a very careful operator, very cool, and can't be excited. He opposes all the known laws of anatomy and physiology, but so did Hunter and others. No one can explain this thing, but it is so. Take a tooth of that kind and scrape off the peridental membrane, implant it, and it will drop out—will not stay in five minutes. All I ask is that you watch this thing, investigate it, and when you get a favorable case try it and see for yourself. Dr. Harper, of St. Louis, has performed the operation, and the case is doing well.

*Dr. Rawls.*—I stand by what I said in the *Southern Journal.* Statements were made by Dr. Younger that were erroneous, and were slurs and reflections on our profession in this country, and according all honor to the profession across the water. Statements were made that can't be proved. I know that in favorable cases the operation can be performed. There is no union in the made socket. The peridental membrane may be as dead as a door nail, and yet be tenacious enough to "cushion" and retain the tooth, but it is not a renewal of life.

[Here the gentlemen challenged one another to proof of the various points in dispute, and the reporter gave it up.]

*Dr. Rawls.*—I admit that the tissues of the tooth may fill up with the fluids of the body, and there is a circulation through it just as in sponge-grafting, but it is only a matter of mechanics—the filling up of space by a fluid mass.

*Dr. C. M. Wright.*—Crowns of natural teeth mounted on plates when in the mouth return to their natural color, but if taken out dry up and lose it. It is not the nutritive fluids that cause the natural appearance—it is simply moisture.

IRREGULARITIES OF THE TEETH.

(See paper by Dr. G. W. Keely, page 161)

*Dr. A. Berry.*—I know of nothing so likely to make a dentist wish he was not a dentist as a difficult and tedious case of regulation, especially if he does not receive adequate compensation, or his labor is not appreciated.

*Dr. C. M. Wright.*—It is almost a specialty within a specialty, and we should have sufficient knowledge on the subject to know how to avoid doing harm. It is not very remunerative, and I think we should all do as I am doing—let somebody else go to the poorhouse for us. I have already recommended several cases to Dr. Keely. He is on the road to the poorhouse, and I am on the road to affluence.

*Dr. W. N. Morrison.*—I must take exception to Dr. Keely's extraction of teeth to get them out of the way. I have never seen a case where there was any benefit from the extraction of the bicuspids or molars. Have sometimes seen cases where they had been extracted which looked all right, and even beautiful, but when you examine them as a whole completed being, they remind us of a person minus a finger. While the occlusion is, apparently, perfect, it cannot be correct occlusion for bicuspids to go between molars, or molars between bicuspids. In my experience, the dentist with his forceps invariably steps in a little too soon. Leave them in, and nature will make room for them. In regulating, we should start with the deciduous teeth. Retain these, even if pulpless. Remove the pulp, even grind away the crowns and leave in the roots to expand the arch. Let the permanent teeth erupt between the roots of the deciduous teeth, thus giving the necessary room by expanding the osseous process. Try one case by extracting where it seems absolutely necessary, and another in the same family by leaving the teeth alone, like the lower animals. The calf or colt does not have teeth extracted to make room for others. You will be surprised at the improvement in the case where the teeth were not extracted. I do not mean to say that I never extract teeth in regulating, but if there was any chance to expand the arch by using that tooth, I should retain it

*Dr. McKellops.*—Is it good dentistry to put a patient in misery for a year or more with complicated regulation appliances? Have we a right to do it? No. If you can regulate them by extracting the teeth that are in the way, do it. You can't get one patient in a thousand to stand it, and few can pay for it. Often after worrying them for months trying to keep in all the teeth, you will be compelled finally to extract.

I have here a little appliance by Dr. Patrick—a 20k gold band. He makes loops of each end around the teeth, to which he anchors and solders the joint. Then each day he crimps the gold band a little, thus drawing or pulling the teeth and holding them there. It is very simple and efficient. I had a case where there were spaces between the teeth, and the teeth were drawn back and together in six weeks, and without pain. In filling I do not depend on quick separations, but use waxed tape and twine to get plenty of room.

*Dr. C. M. Wright.*—To do anything with the present race of people we would have to go back five or six centuries, but we cannot do that, so we would better take the shears and chisel and do the best we can with what we have.

The Relations between Dentistry and Medicine.

(See paper by Dr. Dunn, page 175.)

DISCUSSION.

*Dr. W. A. Dun.*—Hypnotism is not applicable in all cases. [Dr. Dun here introduced a young man, one of the best subjects in Cincinnati. Having hypnotized him, he pierced the back of his hand with a long needle, leaving it in the flesh, the subject being conscious, but feeling no pain. He went through the room showing his hand to each member.] You can influence any part. There is absolute stoppage of nerve sensations being conveyed to the brain.

*Dr. H. A. Smith.*—I have operated for patients under this influence, and there was no pain whatever. The lesion, in one case, was on the labial surface of an incisor, erosion progressing into caries. My first operation was in adjusting an artificial crown.

*Dr. W. H. Fletcher.*—Have used this method in the extraction of roots of teeth successfully.

(Paper by Dr. Harlan on New Remedies, see page 179.)

DISCUSSION.

*Dr. Harlan.*—With reference to obtunders in general, a good deal depends on the manner of the operator in his management of the patient. If any one here present operates all day constantly, he must recognize that some obtudent or medicament is necessary to good or perfect work, and I find that no one obtudent is sufficient, because of idiosyncracies. Absolute alcohol is good in some cases, but the temperature of room being less than the temperature of the body is sufficient to cause exquisite pain. A good many obtundents are increased in efficiency by warming them and drying the cavity with warm air.

As to the empirical use of remedies, how many men are there who know why they use carbolic acid? Our worthy President may know, but take the average dentists as they are distributed through towns and cities and they don't.

An antiseptic is a preservative opposed to putrefaction, but it will not destroy germs. Infection is one thing, and an antiseptic is something else. A disinfectant must destroy the infectious matter—must destroy the spores. How many know that it would take one hundred and fifty pounds of carbolic acid to disinfect this room after a death from scarlet fever, and that one cent's worth of bichloride of mercury would do it better.

Every time a broach, probe, or other instrument is used in the root of a tooth, it should be disinfected before being used again, which will avoid poisoning the socket and causing an abscess. Always fill the carious cavity with eugenol or other disenfectant before opening or entering the pulp chamber, thus preventing the entrance of disease germs.

*Dr. C. M. Wright.*—I have sometimes been successful, with the aid of the patient's imagination, in obtunding sensitive cavities by the use of warm water.

*Dr. J. S. Cassidy.*—In using cocaine, I prefer the following preparation: *Camphor* ʒj, *chloral* ʒj, adding *alkaloid of cocaine* ʒjj and *ether* (*sulph.*) ʒji, which makes an obtundant that will always act.

*Dr. A. O. Rawls.*—Before using an article I want to know what it is we wish to produce. Are we using it as a specific, or mechanically? Are we using it with a full knowledge of its effects upon the tissues, or of the effect we desire to obtain. One would think, to hear the discussions, that an article had some occult or mysterious influence upon the nervous tissues without regard to nature's laws. What we want to know, is how is the effect produced?

Pain is a force, the result of contact. We cannot have pain without disturbance of molecules. We must, after all, take physics for our guide; for instance, alcohol relieves the canaliculi of the watery fluid, leaving nothing in the tubuli to come in contact with the nervous tissues. Anything that prevents pressure will prevent pain. There is nothing but acts through its mechanical aspect. Chemistry is nothing but minute physics. Obtundents simply cause evaporation or distruction of what would convey sensation to the center of feeling. The remedy does nothing but penetrate the moisture; the hard tissues are not the sensitive parts; it is only the movement of the hand on the softer parts that causes the pain.

*Dr. E. G. Betty.*—I notice that the idea running through the discussion is the *modus operandi* of medicines. Cocaine acts because it produces its effects through anæmia. The fact that a bleached appearance and coldness of the part followed the application of the cocaine, brought this idea to my mind. I deny that carbolic acid is an anæsthetic or an obtundent, *per se.* It is an obtundent so far as it penetrates and causes coagulation. The use of alcohol causes death of the parts by rapid and complete abstraction of the water from the dentine. We want to get to rock bottom, and as far as we go we must see, know, and prove.

*Dr. Rawls.*—The same results are produced by either congestion or anæmia.

